# Metagenomic insights into the effects of Chive seed flavonoid on intestinal fermentation, morphology, and microbiota composition in sheep

**DOI:** 10.3389/fmicb.2025.1590400

**Published:** 2025-06-13

**Authors:** Xujie Li, Dengpan Li, Chunhui Wang, Qiao Li, Yajing Nie, Liming Zhang, Jin Xu, Youji Ma

**Affiliations:** ^1^College of Animal Science and Technology, Gansu Agricultural University, Lanzhou, China; ^2^Gansu Key Laboratory of Animal Generational Physiology and Reproductive Regulation, Lanzhou, China

**Keywords:** Chive seed flavonoid, intestinal morphology, VFAs, gut microbiota, sheep

## Abstract

Chive seed flavonoid (CSF) exhibits antioxidant, digestive, and immunomodulatory properties, yet their effects on ruminant intestinal development and microbial ecosystems remain underexplored. This study systematically evaluated CSF supplementation (0.25% of concentrate) on intestinal morphology, volatile fatty acids (VFAs) dynamics, and microbiota composition in 16 weaned Hu lambs (3 months; 19.57 ± 1.56 kg initial weight) using histomorphometry, VFA quantification, and full-length 16S rRNA sequencing. Animals were randomly allocated to a control (basal diet) or CSF-supplemented group (T), following a 7-day adaptation and 90-day experimental protocol. Key findings revealed: (1) The T group demonstrated increased jejunal and ileal villus length (*p* < 0.05), elevated villus height-to-crypt depth ratio (V/C; *p* < 0.05), thickened muscular layers (*p* < 0.05), and reduced crypt depth (*p* < 0.05). (2) CSF supplementation significantly elevated acetate, propionate, butyrate, and total VFAs (*p* < 0.05) in ileum, cecum, colon, and rectum, with notable jejunal propionate and total VFAs increases (*p* < 0.05). (3) Upregulated expression of ileal and cecal genes (*IGF1*, *CD81*, *CTNNBL1*, *SLC39A8*) linked to tissue morphogenesis and VFAs absorption was observed in the T group (*p* < 0.01). (4) Full-length 16S analysis demonstrated CSF-enhanced microbial diversity and selective enrichment of *Cyanobacteriota*, *Bacillus*, *Acetobacterium*, and *Streptomyces* [Linear Discriminant Analysis Effect Size (LEfSe) analysis]. Regional microbial shifts included *Bacteroidota* proliferation in the small intestine and rectum, *Actinomycetota* dominance in duodenum/cecum/colon, and ileal enrichment of *Bacillota*, *Clostridium*, and *Streptomyces*. KEGG pathway analysis confirmed CSF-driven enhancements in carbohydrate/energy metabolism, immune regulation, and intestinal barrier pathways (*p* < 0.05). In conclusion, dietary supplementation with 0.25% CSF improved intestinal morphology, increased the production of VFAs, and optimized microbial composition, thereby promoting intestinal health in sheep. This study provides a theoretical basis for the application of CSF in promoting healthy production in sheep.

## 1 Introduction

The global demand for food safety and the rapid development of the livestock industry have led to the widespread use of antibiotics. However, the long-term administration of antibiotics has resulted in antimicrobial residues and the emergence of antimicrobial resistance, posing significant threats to human health and animal productivity. As a result, there is an urgent need to restrict or ban the use of antibiotics in agriculture (Li et al., 2024). Traditional livestock farming practices are increasingly unable to meet modern demands, driving the sector toward high-quality and sustainable development. To ensure sustainable development, food safety, and public health, the development of alternative feed resources has become a critical area of research (van Dijk et al., 2018). With advancements in biotechnology, multi-omics technologies, particularly microbiome and genomics, have entered the field of animal production. The rise of microbiome research has further promoted sustainable development in animal husbandry, not only improving animal production performance and health but also reducing environmental pollution and enhancing economic efficiency (Liu et al., 2024).

Against this backdrop, Chive seed has garnered attention as a promising agricultural by-product. It contains a wide array of bioactive compounds, including amino acids, flavonoids, and saponins (Sang et al., 2000). Flavonoids, as secondary metabolites widely present in plants, have attracted significant interest in animal nutrition and health due to their notable antioxidant, antimicrobial, anti-inflammatory, and growth-promoting properties (Muqier et al., 2017). Accumulating evidence highlights the diverse benefits of flavonoids in animal health. For instance, soy isoflavones have been found to enhance animal growth while exhibiting anti-obesity and anti-diabetic effects in humans (Nakai and Kamei, 2020). Additionally, flavonoids have been shown to enhance immune function, improve milk and egg production (Amanzadeh et al., 2019), and mitigate conditions such as necrotic enteritis in broilers (Buiatte et al., 2024). Furthermore, these compounds can maintain gut barrier integrity, regulate gut hormone secretion, and modulate the composition and function of gut microbiota (Oteiza et al., 2018). Mulberry leaf flavonoid, for example, have been reported to improve nutrient digestibility and energy utilization in sheep while reducing methane emissions (Chen et al., 2015). CSF, a well-known plant-derived extract, are rich in various phytochemicals, including quercetin, kaempferol, kaempferol glycosides, naringenin, naringenin glycosides, hesperetin, and epicatechin. The total flavonoids present in Allium cepa exhibit strong antioxidant, anti-aging, and antimicrobial effects. These flavonoid compounds are stable in nature, resistant to degradation, and capable of inhibiting food oxidation and suppressing harmful bacteria in food products (de Freitas et al., 2020).

Current research on CSF has primarily focused on their antioxidant, antiviral, and antibacterial effects in humans and mice, with limited studies conducted in ruminants. Ruminant gut microbiota comprise four major phyla: bacteria, fungi, archaea, and protists (Zhang et al., 2024), which play pivotal roles in maintaining gut barriers, enhancing immunity, and promoting metabolism (Hooper and Macpherson, 2010; Zhang et al., 2022). The sheep gut, as a natural barrier, maintains intestinal homeostasis, protects the host against pathogens, and is essential for digestion and metabolism (Lee and Hase, 2014; Li D. et al., 2023; Zhang et al., 2024). A well-functioning gut barrier not only enhances the host’s ability to absorb nutrients but also defends against pathogenic microbial invasions. Based on this background, this study posits that CSF may exert their beneficial effects through their antimicrobial and probiotic properties, modulating gut microbiota composition by significantly increasing the abundance of beneficial bacteria while suppressing the growth of potentially harmful pathogens. This modulation could lead to elevated VFAs levels and improved intestinal pH balance, thereby maintaining gut fermentation homeostasis. Additionally, CSF may enhance gut barrier function and improve intestinal morphological traits, including villus height, muscular layer thickness, and crypt depth optimization, ultimately improving nutrient absorption efficiency. To explore these hypotheses, this study investigates the effects of dietary supplementation with 0.25% CSF on gut tissue morphology, microbial composition, and fermentation parameters in sheep. These findings aim to provide theoretical support for the application of CFS in ruminant nutrition.

## 2 Materials and methods

### 2.1 Ethic statement

All experimental procedures were conducted in accordance with the guidelines of the Ministry of Science and Technology of the People’s Republic of China (Approval No. 2006-398) and approved by the Animal Ethics and Welfare Committee of Gansu Agricultural University (GSAU-AEW-2020-0057).

### 2.2 Animals, feeding management and experimental design

This experiment adopted a completely randomized single-factor design. Sixteen Hu lambs (3-month-old; initial body weight: 19.56 ± 1.59 kg) with homogeneous genetic backgrounds and comparable health status were selected as experimental subjects. The lambs, sourced from Shengyuan Agricultural and Animal Husbandry Technology Co., Ltd. (Linxia Hui Autonomous Prefecture, Gansu Province, China), were randomly allocated into two groups (8 lambs/group): a control group (CK) fed a basal diet and an experimental group (T) receiving the basal diet supplemented with 0.25% CSF in the concentrate portion. The trial included a 7-day adaptation period and a 90-day experimental period. The basal diet was formulated in accordance with the Chinese agricultural standard *Nutrient Requirements of Meat-Type Sheep and Goats* (NY/T 816-2021), with detailed compositions and nutritional levels provided in Table 1, while CSF’s nutritional and bioactive profiles are presented in Table 2. All lambs were housed individually in pens within a disinfected facility. Prior to the trial, animals were ear-tagged for identification and acclimatized to the environment. Daily feeding occurred at 07:00 and 18:00 h, with *ad libitum* access to feed and water. Pens were cleaned daily to remove feces and urine, and routine disinfection was performed to maintain hygienic conditions.

**TABLE 1:** Composition and nutritional levels of the basal diet (dry matter basis).

Diet ingredient	Content/%
Corn	33.00
Corn germ meal	6.00
Soybean meal	12.00
Cottonseed meal	6.00
Whole corn silage	22.00
Alfalfa hay	14.00
Wheat bran	4.00
Sodium bicarbonate	0.90
Limestone	1.20
Salt	0.60
Premix1	0.30
Total	100.00
**Nutritional levels**	
Metabolic energy (MJ/kg)	11.52
Crude protein (%)	17.23
Crude fat (%)	3.14
NDF (%)	25.60
ADF (%)	16.14
Ga (%)	0.83
P (%)	0.42
Dry matter (%)	89.2

NDF, neutral detergent fiber; ADF, acid detergent fiber. The same as the table below. The premix provided per kg of diet: Fe 100 mg, Cu 8.5 mg, Zn 65 mg, Se 0.25 mg, I 0.5 mg, Co 0.30 mg, Mn.40 mg, VA 10,000 IU, VD 5,000 IU, VE 150 IU. ME was a calculated value, while the others were measured values.

**TABLE 2:** Composition and nutritional levels of Chive seed flavonoid (dry matter basis).

Items	Content/%	Items	Content/%
Crude protein	16.82	Ca	0.52
Crude fat	14.62	P	0.68
NDF	35.70	Dry matter	92.01
ADF	24.55	Total flavonoids	0.5

### 2.3 Sample collection

At the conclusion of the trial, all sheep underwent a 12-h fasting period followed by 2 h of water deprivation. Five lambs per group were randomly selected and euthanized via intravenous injection 30 mL of 20% pentobarbital sodium solution. Immediately post-euthanasia, the abdominal cavity was opened, and junctions between intestinal segments (duodenum, jejunum, ileum, cecum, colon, rectum) were ligated to prevent cross-contamination of luminal contents. Luminal contents from the mid-sections of each segment were filtered through four-layer gauze into pre-labeled beakers for pH measurement. Aliquots of the solid-liquid mixtures were transferred into 5 mL cryotubes, flash-frozen in liquid nitrogen, and transported to the laboratory within 1 hour for storage at –80°C pending VFAs and microbial community analyses. Concurrently, two 1 cm^2^ tissue samples were excised from the mid-portions of the duodenum, jejunum, and ileum. These tissues were rinsed three times with sterile phosphate-buffered saline (PBS; pH 7.4) to remove residual luminal contents, one of the rinsed samples followed by immersion fixation in 4% paraformaldehyde (4°C, 24–48 h) to preserve histological integrity, and the other was placed in a cryopreservation tube and filled with liquid nitrogen for subsequent quantitative analysis.

### 2.4 Morphological indicators of small intestinal tissue

Intestinal tissues fixed in a 4% paraformaldehyde solution were subjected to dehydration, clearing, and embedding processes to create paraffin sections with a thickness of 6 μm. These sections were stained with hematoxylin-eosin (HE) and examined under a microscope at 10x magnification. A digital slide scanner (Pannoramic DESK, Hungary) was utilized to scan the sections, and the Slide Viewer 2.6 image analysis system was employed to analyze three discontinuous sections per sample. Measurements of villus height, crypt depth, and muscle layer thickness in the duodenum, jejunum, and ileum were conducted, with at least five fields of view measured per section. The average values were subsequently calculated to provide the final measurement data.

### 2.5 Determination of fermentation parameters in intestinal contents

The pH levels of the contents in each intestinal segment were measured using a portable pH meter (LC23020970, Shanghai, China). The VFAs present in the contents of each intestinal segment were quantified following the gas chromatography method outlined in previous studies (Shi et al., 2023). Briefly, the contents of each intestinal segment were thawed in a refrigerator at 4°C, and 1 g of cecal, colonic, and jejunal contents was dissolved in 2 mL of pure water. Subsequently, the contents from all six intestinal segments were transferred into a 5 mL centrifuge tube and centrifuged at 5,400 rpm for 10 minutes at 4°C. After centrifugation, 1 mL of the supernatant was transferred into a 1.5 mL centrifuge tube, to which 0.2 mL of a 25% metaphosphoric acid deproteinization solution (containing 2 g/L of the internal standard 2EB) was added. The mixture was thoroughly mixed and incubated on ice for 30 min. Following this, it was centrifuged at 10,000 rpm for 10 min at 4°C. The supernatant was then drawn using a disposable syringe, filtered through a 0.22 μm membrane into a 2 mL brown sample vial, and the VFAs were measured using a gas chromatograph (HP6890N, Agilent, United States) equipped with an HP19091N-213 capillary column (Agilent, United States).

### 2.6 16S rRNA Full-length sequencing of intestinal microbiota

#### 2.6.1 DNA extraction and quality control

Total genomic DNA samples were extracted using the OMEGA Soil DNA Kit M5635-02 (Omega Bio-Tek, Norcross, GA, United States) following the manufacturer’s instructions and stored at –20°C prior to further analysis. The quantity and quality of the extracted DNA were assessed using the NanoDrop NC 2000 spectrophotometer (Thermo Fisher Scientific, Waltham, MA, United States) and agarose gel electrophoresis, respectively.

#### 2.6.2 PCR amplification and library construction

Following DNA extraction, PCR amplification of the full-length 16S rRNA gene, which encompasses the V1-V9 regions of bacteria, was conducted using standard bacterial 16S full-length primer sequences (27F: AGRGTTYGATYMTGGCTCAG, 1492R: RGYTACCTTGTTACGACT). All PCR amplification products were purified with Amplecourt AMPure Beads (Beckman Coulter, Indianapolis, IN) and subsequently quantified using the PicoGreen dsDNA Assay Kit (Invitrogen, Carlsbad, CA, United States). Library construction was performed utilizing the TruSeq Nano DNA LT Library Prep Kit (SMRT technology) from Illumina (San Diego, CA, United States). After rigorous quality control, the qualified libraries were dispatched to Personal Biotechnology Co., Ltd. (Shanghai, China) for sequencing on the PacBio Sequel II platform.

### 2.7 Sequencing data analysis

The raw sequencing data obtained from high-throughput sequencing undergoes an initial screening based on sequence quality, with problematic samples being subjected to re-sequencing or supplementary sequencing. The raw sequences that pass this initial quality screening are then assigned to libraries and samples according to their Index and Barcode information, followed by the removal of barcode sequences. Subsequently, in accordance with the methodology (Bolyen et al., 2019), the sequences undergo quality filtering, denoising, operational taxonomic unit (OUT) clustering, merging, and chimera removal using the dada 2 plugin of the QIIME 2 software. The sequence data analysis is primarily conducted using QIIME 2 and the R package (v3.2.0). Based on the distribution of ASV/OTU across different samples, the Alpha diversity levels of each sample are evaluated, and the adequacy of the sequencing depth is assessed through the rarefaction curve. Further evaluation of Alpha diversity indices such as Chao 1, Observed-species, Faith-pd, Goods-coverage, Shannon, and Simpson is performed. By plotting the rank abundance curves at the ASV level, the richness and evenness of ASVs across different samples are compared. At the ASV/OTU level, R software (v3.2.0) is utilized to generate Principal Coordinate Analysis PCoA and Non-metric Multidimensional Scaling NMDS plots, combined with statistical test methods (such as ANOSIM) to quantify the differences in Beta diversity between different groups and assess their significance. Finally, statistical analysis is conducted on the feature table after the removal of singletons, allowing for the visualization of the compositional distribution of each sample at the phylum and genus taxonomic levels, with the analysis results presented in bar charts Utilize LEfSe to identify differentially abundant taxa among various groups, and represent significantly enriched species along with their importance levels within each group through a bar graph illustrating the distribution of LDA values for these significant species. Conduct functional predictions by analyzing 16S rRNA gene sequences in the KEGG database using PICRUSt 2, and employ STAMP v2.1.3 software to visualize differences in gene enrichment across metabolic pathways among distinct samples or groups.

### 2.8 Quantitative real-time PCR

To elucidate the potential causal relationships between microbiota-host interactions identified through 16S rRNA full-length sequencing data, this study focused on the ileum and cecum segments of sheep, which showed the most pronounced responses to CSF intervention, as evidenced by significant changes in intestinal histomorphology and fermentation parameters. Based on prior microbiota analysis suggesting their relevance to host-microbe crosstalk, mucosal epithelial tissues from these segments were subjected to quantitative real-time PCR (qPCR) analysis of six candidate genes (*IGF1*, *CD81*, *CTNNBL1*, *MGST1*, *GPX4*, *SLC39A8*) implicated in histomorphological development and VFAs sorption. Total RNA in intestinal tissues was extracted using TRIzol reagent (Beijing Quanshijin Biotechnology Co., Ltd., China), the concentration and purity of total RNA were detected using an ultramicro spectrophotometer (Implen, Germany), and then according to the Evo-MLV RT kit (Accurate Biotechnology, Hunan) Reverse transcribe cDNA from the isolated total RNA in China, followed by qPCR amplification with the SYBR Green qPCR Kit from the same manufacturer. Each experiment included at least four independent biological replicates. Primer sequences are detailed in Table 3. Gene expression levels were normalized to the β-actin reference gene and quantified using the 2-ΔΔCt method.

**TABLE 3:** Primer sequence information.

Gene	Primer sequence(5′–3′)	Length	Annealing temperature	Accession number
β-Actin	F:AGCCTTCCTTCCTGGGCATGGA R:GGACAGCACCGTGTTGGCGTAGA	113 bp	60°C	NM_001009784.3
IGF1	F:AGGAATCGTGGATGAGTGCTG R:ACTCCCTCTGCTTGTGTTCTTC	169 bp	60°C	XM_060411609.1
CD81	F:ATGACCCACAGACCACCAAC R:ACTCCCTCTGCTTGTGTTCTTC	127 bp	60°C	NM_001127281.1
CTNNBL1	F:CCCTGGTGAAGATGCTTGGT R:CGCACTGCCATTTTAGCTCC	106 bp	60°C	NM_001308590.1
MGSTI	F:CCTTTGCCTCTTACACGACG R:CACCCTGTCATCTGTCCGAA	164 bp	60°C	XM_004006818.5
GPX4	F:AAGAACGGCTGTGTGGTGAAG R:AGGGTGAGGGACGACTTTTCC	106 bp	60°C	XM_060416444.1
SLC39A8	F:TCGCCTGGATGATAACGCTC R:TGTCGAGTGCTCATTCCTGC	184 bp	60°C	XM_060416847.1

### 2.9 Statistical analysis

The experimental data were initially organized using Excel 2024 software, and the differences in the data were analyzed using SPSS 25.0 (version 25.0, SPSS Inc., Chicago, IL, United States). Independent sample *t*-tests were conducted to analyze the pH and VFAs content across different intestinal segments of the two groups of lambs, with results presented as means and standard errors of the mean (SEM). 0.05 ≤ *p* < 0.1 was considered indicative of a trend, *p* < 0.05 was deemed a significant difference, and *p* < 0.01 was classified as a highly significant difference, all of which are statistically meaningful. Furthermore, Spearman’s correlation test was employed to analyze the correlation between intestinal microbiota and fermentation parameters; r < 0 means negative correlation, r > 0 means positive correlation.

## 3 Results

### 3.1 Effects of Chive seed flavonoid on intestinal fermentation parameters, intestinal barrier function and small intestinal tissue morphology in sheep

The CK group exhibited significantly elevated pH values and total TVFAs concentrations in the duodenum and jejunum compared to the T group (*p* < 0.05), as detailed in Table 4. Conversely, the butyric acid content in the T group was highly significant that in the CK group (*p* < 0.01). In the ileum, colon, and rectum segments (Tables 4, 5), the pH value, acetic acid, propionic acid, butyric acid, and TVFAs content in the T group were significantly higher than those in the CK group (*p* < 0.05). Notably, in the cecum, the T group exhibited significantly greater concentrations of acetic acid, propionic acid, and TVFAs compared to the CK group (*p* < 0.01). As illustrated in Table 6), the villus length and muscularis thickness in the jejunum and ileum of the T group were significantly higher than those of the CK group (*p* < 0.01). The crypt depth in the CK group was significantly higher than that in the T group in the jejunum (*p* < 0.05) and highly significantly higher in the duodenum and ileum (*p* < 0.01). Additionally, the villus length in the duodenum of the CK group was significantly higher than that of the T group (*p* < 0.01), while V/C ratio in the jejunum and ileum of the T group was significantly higher than that of the CK group (*p* < 0.01), with no significant difference between the two groups in the duodenum (*p* > 0.05). In the cecum, the T group exhibited significantly greater concentrations of acetic acid, propionic acid, and TVFAs compared to the CK group (*p* < 0.01).

**TABLE 4:** Effects of Chive seed flavonoid on small intestinal fermentation parameters in sheep.

Region	Items	Group		SEM	*P*-value
		CK	*T*		
Duodenum	pH	6.56^a^	6.49^b^	0.017	0.028
	Acetate (mmol/L)	15.91	14.77	0.325	0.066
	Propionate (mmol/L)	4.13	3.64	0.153	0.110
	Butyrate (mmol/L)	3.17	2.84	0.124	0.212
	Acetate/propionate	3.86	4.08	0.126	0.437
	TVFAs (mmol/L)	26.14^a^	23.69^b^	0.626	0.022
Jejunum	pH	6.85^a^	6.13^b^	0.162	<0.001
	Acetate (mmol/L)	15.53	14.44	0.369	0.153
	Propionate (mmol/L)	3.68^b^	3.78^a^	0.268	0.028
	Butyrate (mmol/L)	2.79^a^	1.61^b^	0.268	<0.001
	Acetate/propionate	4.22	3.86	0.180	0.150
	TVFAs (mmol/L)	26.39^a^	21.53^b^	1.132	0.002
Ileum	pH	6.88^b^	7.04^a^	0.038	<0.001
	Acetate (mmol/L)	23.56^b^	26.82^a^	0.758	0.002
	Propionate (mmol/L)	5.27^b^	6.55^a^	0.317	0.013
	Butyrate (mmol/L)	1.49^b^	4.04^a^	0.573	<0.001
	Acetate/propionate	4.48	4.10	0.107	0.54
	TVFAs (mmol/L)	31.44^b^	41.78^a^	2.371	0.001

CK, the control group; T, the experimental group; SEM, standard error of the mean; TVFAs, total volatile fatty acids. The two groups were compared; Numerical superscripts without letters indicate no significant difference, while different letters indicate significant difference. The same as the table below.

**TABLE 5:** Effects of Chive seed flavonoid on large intestinal fermentation parametersin in sheep.

Region	Items	Group		SEM	*P*-value
		CK	*T*		
Cecum	pH	7.00	7.02	0.011	0.602
	Acetate (mmol/L)	53.08^b^	64.76^a^	2.648	<0.001
	Propionate (mmol/L)	11.51^b^	16.59^a^	1.206	0.006
	Butyrate (mmol/L)	3.59	4.26	0.246	0.202
	Acetate/propionate	4.16^a^	3.92^b^	0.180	0.029
	TVFAs (mmol/L)	70.11^b^	87.82^a^	4.088	0.001
Colon	pH	7.09^b^	7.19^a^	0.025	0.013
	Acetate (mmol/L)	24.38^b^	39.21^a^	3.318	<0.001
	Propionate (mmol/L)	5.70^b^	8.41^a^	0.691	0.022
	Butyrate (mmol/L)	2.41^b^	3.37^a^	0.246	0.023
	Acetate/propionate	4.46	4.66	0.180	0.755
	TVFAs (mmol/L)	33.72^b^	52.75^a^	4.280	<0.001
Rectum	pH	7.11	7.15	0.009	0.035
	Acetate (mmol/L)	10.97^b^	22.87^a^	2.676	<0.001
	Propionate (mmol/L)	2.76^b^	5.29^a^	0.564	<0.001
	Butyrate (mmol/L)	1.04^b^	1.70^a^	0.161	0.010
	Acetate/propionate	3.98^b^	4.33^a^	0.090	0.016
	TVFAs (mmol/L)	15.88^b^	33.96^a^	4.059	<0.001

**TABLE 6:** Effects of Chive seed flavonoid on small intestinal histomorphology in sheep.

Region	Items	Group		SEM	*P*-value
		CK	*T*		
Duodenum	Villus height/μM	535.51^a^	435.18^b^	16.470	<0.001
	Crypt depth/μM	381.88^a^	261.23^b^	22.247	0.001
	Muscular layer thickness/μM	130.56	132.96	3.243	0.729
	V/C	1.42	1.69	0.074	0.066
Jejunum	Villus height/μM	418.44^b^	467.27^a^	9.866	0.005
	Crypt depth/μM	338.26^a^	300.85^b^	9.425	0.04
	Muscular layer thickness/μM	112.36^b^	157.39^a^	6.944	<0.001
	V/C	1.24^b^	1.56^a^	0.024	0.007
Ileum	Villus height/μM	373.10^b^	551.10^a^	27.510	<0.001
	Crypt depth/μM	315.10^a^	232.27^b^	28.116	<0.001
	Muscular layer thickness/μM	101.13^b^	185.73^a^	13.319	<0.001
	V/C	1.18^b^	2.37^a^	0.057	0.005

V/C, Villus height/Crypt depth.

**FIGURE 1:**
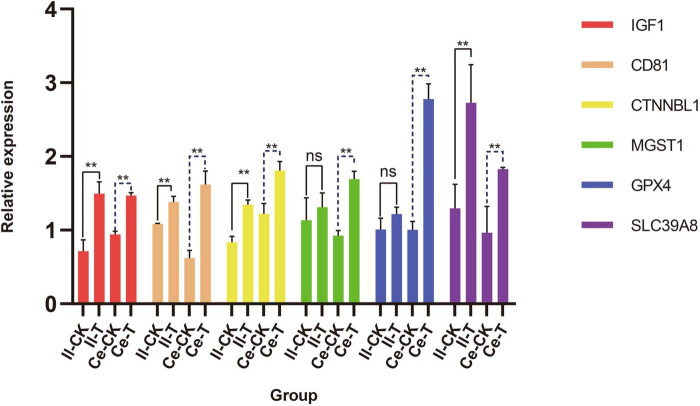
Quantitative analysis of genes associated with the regulation of tissue morphogenesis and VFAs absorption in the ileum and cecum. Data are expressed as mean ± SD. Significance levels: **p < 0.01; ns, not significant. The letters Il and Ce represent ileum and cecum.

Quantitative analysis of six candidate genes potentially involved in tissue morphogenesis regulation and VFAs absorption (Figure 1) revealed distinct expression patterns between ileum and cecum epithelia. In the ileum, epithelial barrier-associated genes *IGF1*, *CD81*, *CTNNBL1*, and *SLC39A8* exhibited differential expression, with highly significant relative expression levels in T group versus CK group (*p* < 0.01). While *MGST1* and *GPX4* showed an upward trend in T group compared to CK, these differences lacked statistical significance (*p* > 0.05). In contrast, cecum analysis demonstrated markedly elevated relative expression levels of *IGF1*, *CD81*, *CTNNB2*, *MGST1*, *GPX4*, and *SLC39A8* in T group relative to CK group (*p* < 0.01).

### 3.2 Effects of Chive seed flavonoid on the whole of intestine microbiota in sheep

**FIGURE 2:**
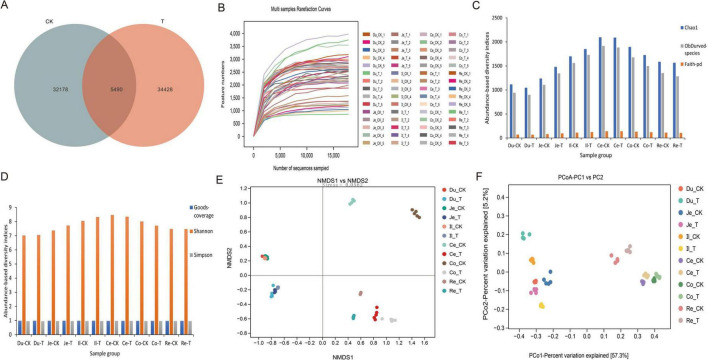
Effects of Chive seed flavonoid on Gut Microbial Diversity in Sheep. **(A)** OTU distribution. **(B)** Rarefaction curves. **(C,D)** Alpha diversity indices (Chao1, observed species, Faith-pd, Goods-coverage, Shannon, Simpson). **(E)** NMDS analysis of beta diversity. **(F)** PCoA analysis of beta diversity. The letters Du, Je, Il, Ce, Co, and Re represent the duodenum, jejunum, ileum, cecum, colon, and rectum, respectively. The same applies to the figure below.

This study utilized the Vsearch software to match the upstream and downstream primers for the full-length 16S rRNA V1-V9 region sequencing raw data, resulting in a total of 3,996,732 sequences. After denoising and clustering, chimeras were removed, yielding 3,103,668 high-quality sequences. Subsequently, the FrameBot functional gene project was employed for correction, and singletons were excluded, ultimately producing 2,333,782 sequences, with an average of 38,896 sequences per sample (Supplementary Table 1). Using the Venn Diagram software for clustering and reading data, we analyzed the number of features between the two groups and found that the total number of features in the CK group in the sheep intestine was 32,178, while the T group had 34,428 features, with 5,490 OTUs shared between them (Figure 2A). The Rarefaction Curve indicated that the curves for the 60 samples tended to flatten as the number of sequences increased, suggesting that the sequencing results adequately reflected the diversity of the current samples, and further increasing the sequencing depth would not uncover a significant number of previously undiscovered new ASVs/OTUs (Figure 2B).

This study conducted an Alpha diversity analysis of the intestinal microbiota in two groups of sheep, with results presented in Figures 2C,D and Table 7. Specifically, the Chao1 index, Observed-species index, Faith-pd index, Shannon index, and Simpson index were all higher in the T group compared to the CK group; however, these differences did not reach statistical significance (*p* > 0.05). Additionally, the Goods-coverage index showed no significant difference between the two groups (*p* > 0.05). To further investigate the distribution differences in microbial communities between the sample groups, we employed NMDS and PCoA for Beta diversity analysis. PCoA is an analytical method based on a linear model, while NMDS employs a non-linear model. The results of the NMDS analysis (Figure 2E) indicate no significant differences in microbial communities among samples within groups (*p* > 0.05), while significant distributional differences were observed between samples across groups (*p* < 0.05). Furthermore, the PCoA results (Figure 2F) reveal that the distances between samples within groups are relatively close, indicating significant differences between groups (*p* < 0.05).

**TABLE 7:** Differences in alpha diversity indices of gut microbiota between CK and T groups.

Items	Group		SEM	*P*-value
	CK	T		
Chao1	2235.745	2429.182	88.803	0.225
Observed-species	1946.547	2090.293	80.040	0.374
Faith-pd	123.835	135.481	3.685	0.115
Goods-coverage	0.973	0.970	0.001	0.389
Shannon	8.697	9.075	0.150	0.210
Simpson	0.980	0.988	0.002	0.051

### 3.3 Effects of Chive seed flavonoid on the composition of intestine microbiota in sheep

**FIGURE 3:**
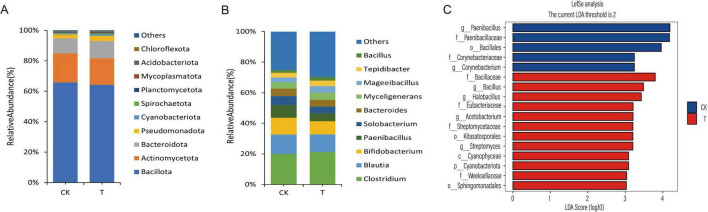
Analysis of differences in gut microbiota composition in sheep influenced by Chive seed flavonoid. **(A)** Phylum-level composition of gut microbiota between CK and T groups. **(B)** Genus-level composition of gut microbiota between CK and T groups. **(C)** LEfSe analysis showing LDA values for differentially abundant biomarkers.

The visualization results revealed that at the phylum level (Figure 3A), six groups—Bacteroidota, Pseudomonadota, Cyanobacteriota, Spirochaetota, Planctomycetota, and Acidobacteriota—were dominant in the T group, with their proportions being 64.17, 3.39, 0.93, 0.73, 0.56, and 0.23%, respectively, all of which were higher than those in the CK group. In contrast, four groups—Bacillota, Actinomycetota, Mycoplasmatota, and Chloroflexota—dominated in the CK group, accounting for 65.83, 19.07, 0.42, and 0.21%, respectively, all of which were higher than those in the T group. At the genus level (Figure 3B), among the top 10 bacterial genera in the gut microbiota, five genera—*Clostridium, Myceligenerans, Mageeibacillus, Tepidibacter* and *Bacillus*—were the dominant microbiota in the T group, with proportions of 21.25, 4.72, 4.23, 3.61, and 2.34%, respectively, all of which were higher than those in the CK group. The genera *Blautia, Bifidobacterium, Paenibacillus, Solobacterium* and *Bacteroides* were dominant in the CK group, with proportions of 12.66, 11.09, 8.14, 5.92, and 4.96%, respectively, all of which were higher than those in the T group. Based on cluster analysis, we conducted LEfSe analysis (LDA > 3), which revealed a total of 17 statistically significant biomarkers. These biomarkers are distributed across various taxonomic levels: 1 at the phylum level, 1 at the class level, 3 at the order level, 6 at the family level, and 6 at the genus level. In the T group, 12 biomarkers, including Cyanobacteriota, *Bacillus, Halobacillus*, *Acetobacterium* and *Streptomyces*, were identified. In contrast, the CK group yielded 5 biomarkers, such as *Paenibacillus*, *Paenibacillaceae*, and *Corynebacterium*, as illustrated in Figure 3C.

### 3.4 Effect of Chive seed flavonoid on the intestinal microbiota of sheep small intestine

**FIGURE 4:**
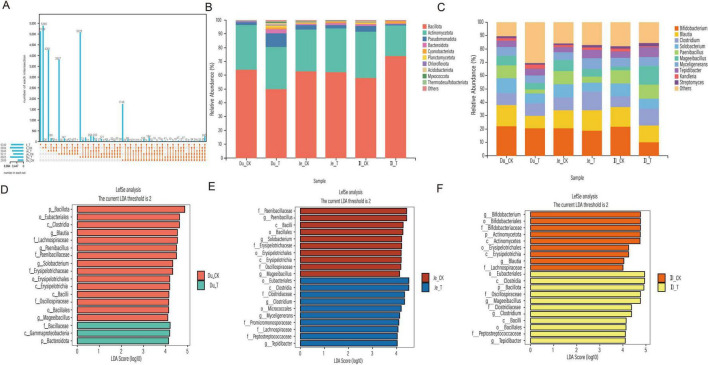
Analysis of the small intestinal microbial community in sheep. **(A)** UpSet plot showing the number of microbial features. **(B)** Relative abundance of microbial phyla. **(C)** Genus-level relative abundance. **(D)** LDA distribution histogram of OTUs in the duodenum. **(E)** LDA distribution histogram of OTUs in the jejunum. **(F)** LDA distribution histogram of OTUs in the ileum.

Analysis of the small intestine microbiota revealed notable differences in microbial composition between the CK and T groups. A total of 245 shared OTUs were identified across both groups. In the duodenum and jejunum, the T group exhibited a higher number of microbial features compared to the CK group. However, in the ileum, the CK group exhibited a higher number of microbial features. Overall, the total number of microbial features across the small intestine segments followed the order: ileum > jejunum > duodenum (Figure 4A). At the phylum level (Figure 4B), Bacillota and Actinomycetota were the dominant phyla in both groups across all intestinal segments, accounting for more than 80% of the total composition. Other phyla, such as Pseudomonadota and Bacteroidota, were also present in relatively high proportions. A detailed comparison revealed that in the duodenum, only Bacillota (63.94% in CK vs. 49.80% in T) and Actinomycetota (32.61% in CK vs. 30.82% in T) were less abundant in the T group compared to the CK group. Notably, Pseudomonadota exhibited the largest difference between the two groups (9.91% in T vs. 1.84% in CK). In the jejunum, only Actinomycetota (31.54% in T vs. 30.15% in CK), Bacteroidota (1.24% in T vs. 1.03% in CK) and Cyanobacteriota (0.94% in T vs. 0.37% in CK) were more abundant in the T group compared to the CK group. In the ileum, only Bacillota (73.91% in T vs. 57.94% in CK) and Cyanobacteriota (1.75% in T vs. 0.32% in CK) were more abundant in the T group compared to the CK group. At the genus level (Figure 4C), *Bifidobacterium* and *Blautia* were the most abundant genera in both groups, accounting for more than 20% of the total composition. In the duodenum, *Clostridium* (9.29% in T vs. 8.83% in CK) and *Kandleria* (3.94% in T vs. 1.78% in CK) exhibited higher relative abundances in the T group compared to the CK group. In the jejunum, *Blautia* (15.39% in T vs. 13.28% in CK), *Clostridium* (14% in T vs. 9.68% in CK), *Myceligenerans* (8.15% in T vs. 5.53% in CK), *Tepidibacter* (6.12% in T vs. 4.02% in CK), *Kandleria* (1.76% in T vs. 1.54% in CK) and *Streptomyces* (1.91% in T vs. 1.14% in CK) were significantly more abundant in the T group compared to the CK group. In the ileum, *Clostridium* (12.64% in T vs. 7.90% in CK), *Paenibacillus* (10.57% in T vs. 10% in CK), *Mageeibacillus* (14.19% in T vs. 2.67% in CK), *Myceligenerans* (6.59% in T vs. 6.51% in CK), *Tepidibacter* (7.33% in T vs. 4.84% in CK) and *Streptomyces* (2.44% in T vs. 1.83% in CK) exhibited higher relative abundances in the T group compared to the CK group.

To further explore differences between groups, LEfSe analysis (LDA > 4) was performed. In the duodenum, 18 differential biomarkers were identified (Figure 4D), in the T group, *Bacteroidota*, *Gammaproteobacteria* and Bacillaceae were significantly enriched, while in the CK group, Bacillota, Eubacteriales, Clostridia and Blautia were significantly enriched. In the jejunum, 20 differential biomarkers were identified (Figure 4E), in the T group, *Clostridium*, *Myceligenerans and Tepidibacter* were significantly enriched, while in the CK group, *Paenibacillus*, *Solobacterium* and *Mageeibacillus* were significantly enriched. In the ileum, 20 differential biomarkers were identified (Figure 4F), in the T group, Bacillota, *Mageeibacillus, Clostridium* and *Tepidibacter* were significantly enriched, while in the CK group, Actinomycetota, *Bifidobacterium* and *Blautia* were significantly enriched. These findings demonstrate that the addition of CSF significantly altered the structure of the small intestinal microbiota.

### 3.5 Effect of Chive seed flavonoid on the intestinal microbiota of sheep large intestine

**FIGURE 5:**
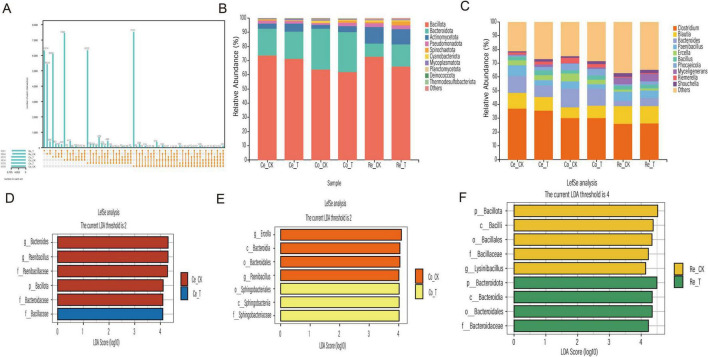
Analysis of the large intestinal microbial community in sheep. **(A)** UpSet plot showing the number of microbial features. **(B)** Relative abundance of microbial phyla. **(C)** Genus-level relative abundance. **(D)** LDA distribution histogram of OTUs in the blind sac. **(E)** LDA distribution histogram of OTUs in the colon. **(F)** LDA distribution histogram of OTUs in the rectum.

The gut microbiota composition of sheep was analyzed using 16S rRNA amplicon sequencing to investigate the effects of CSF. After quality control, the unique OTUs and shared OTUs across blind sac, colon, and rectum habitats were identified. The CK group exhibited 7,513 unique OTUs in the blind sac, 7,461 in the colon, and 5,424 in the rectum. In contrast, the T group had 6,322 unique OTUs in the blind sac, 6,092 in the colon, and 6,314 in the rectum. Notably, the CK group had higher unique OTU counts in the blind sac and colon compared to the T group, but lower unique OTU counts in the rectum. The shared OTUs among all groups totaled 123, as shown in the UpSet plot (Figure 5A). This analysis further confirmed that CSF significantly influenced the gut microbiota structure in sheep. Statistical analysis of the filtered OTU table revealed significant microbiota diversity changes at the phylum level (Figure 5B). Bacillota and Bacteroidota were the most abundant phyla in the sheep gut microbiota, collectively accounting for over 80% of the community. Actinomycetota, Pseudomonadota, and Spirochaetota followed, collectively exceeding 4%. Compared to the CK group, the T group exhibited higher relative abundances of Bacteroidota (19.17% vs. 18.82%) and Actinomycetota (5.54% vs. 3.36%). Additionally, Pseudomonadota and Spirochaetota abundances were higher in the CK group in the blind sac but higher in the T group in the colon and rectum. Cyanobacteriota, Mycoplasmatota, and Planctomycetota abundances were higher in the T group in the blind sac but lower in the colon and rectum. At the genus level (Figure 5C), significant differences in microbial abundances were observed across groups. *Clostridium* dominated the gut microbiota, comprising over 25% of the community. *Blautia, Bacteroides* and *Paenibacillus* followed, collectively exceeding 20%. Compared to the CK group, the T group showed higher abundances of *Blautia* in the colon and Bacteroides in the rectum. Additionally, *Phocaeicola, Myceligenerans* and *Riemerella* abundances were higher in the T group’s blind sac and rectum compared to the CK group, while *Ercella, Bacillus and Shouchella* were more abundant in the T group’s blind sac and colon.

Further analysis using LEfSe revealed significant differences in microbial biomarkers (LDA > 4) between groups. In the blind sac, six discriminant species were identified, with *Bacillaceae* being the only taxon at the family level with higher abundance in the T group (Figure 5D). In the colon, seven discriminant species were identified, including *Sphingobacteriales*, *Sphingobacteriia*, and *Sphingobacteriaceae* at the order, class, and family levels, which were more abundant in the T group. *Ercella* and *Paenibacillus* were identified as abundant species at the genus level in the CK group (Figure 5E). In the rectum, nine discriminant species were identified, with *Bacteroidota* being the abundant phylum in the T group and Bacillota dominating in the CK group (Figure 5F). These findings demonstrate that the addition of CSF significantly improved the gut microbiota community structure in sheep.

### 3.6 Intestinal microbial gene functional prediction, metagenomic analysis of microbial community, fermentation parameters, and intestinal tissue morphology correlation

#### 3.6.1 Prediction of small intestinal microbial gene functional profiles, metagenomic analysis of microbial community, fermentation parameters, and intestinal tissue morphology correlation

**FIGURE 6:**
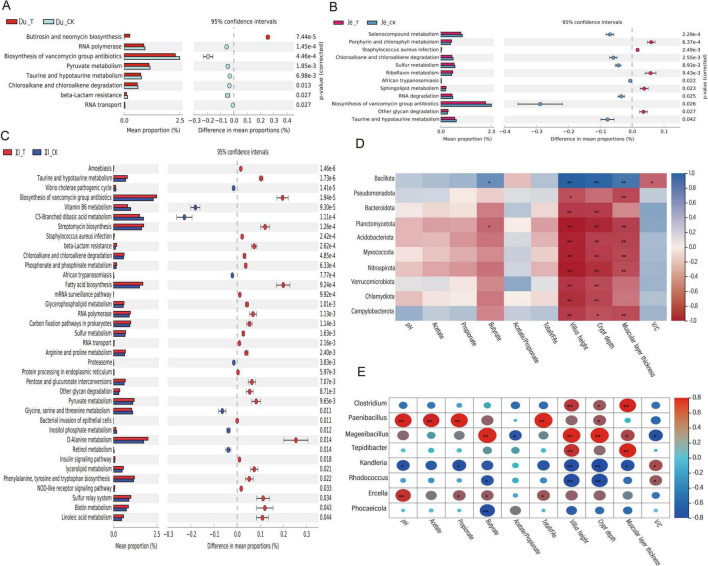
KEGG Pathway and Interaction Correlation Heatmap. **(A)** KEGG functional prediction analysis of microbial communities in the duodenum. **(B)** KEGG functional prediction analysis of microbial communities in the colon. **(C)** KEGG functional prediction analysis of microbial communities in the jejunum. **(D)** Correlation heatmap between small intestinal microbiota and fermentation parameters/organ morphology (top 20 genus-level microbial communities). **(E)** Correlation heatmap between small intestinal microbiota and fermentation parameters/organ morphology (top 20 phylum-level microbial communities). Adjusted *p-value < 0.05 and **p < 0.01 by Student’s *t*-test.

To predict the functional profiles of microbial genes, we employed the PICRUSt 2 software (based on 16S rRNA full-length sequencing) to infer gene functions from the microbial community. A total of 57 KEGG Orthology families were identified, encompassing pathways related to elements, amino acids, secondary metabolite biosynthesis, nucleic acids, lipids, vitamins, and protein transport. In the duodenum, 8 KEGG pathways exhibited significant differences (*p* < 0.05) (Figure 6A). Compared to the CK group, the addition of CSF significantly enriched the butirosin and neomycin biosynthesis pathways (*p* < 0.01). In the jejunum, 12 KEGG pathways showed significant differences (*p* < 0.05) (Figure 6B). These included porphyrin and chlorophyll metabolism, riboflavin metabolism, sphingolipid metabolism, and other glycan degradation pathways, which were significantly enriched in the T group compared to the CK group (*p* < 0.05). Conversely, the CK group exhibited significantly higher enrichment of selenium-containing compound metabolism and chloroalkane and chloroalkene degradation pathways compared to the T group (*p* < 0.05). In the ileum, 37 KEGG pathways were detected (Figure 6C). Among these, 29 pathways (e.g., biosynthesis of vancomycin group antibiotics, streptomycin biosynthesis, fatty acid biosynthesis, and D-Alanine metabolism) exhibited significantly higher enrichment in the T group compared to the CK group (*p* < 0.05). Overall, the KEGG pathways significantly associated with the predicted gene profiles were primarily enriched in metabolic pathways, immune regulation, and energy supply-related physiological responses.

To investigate the correlation between gut microbiota, fermentation parameters, and gut tissue morphology, we selected the 20 most abundant microbial communities at the phylum and genus levels using the Spearman correlation analysis method. We then analyzed their relationships with fermentation parameters and gut tissue morphology. After excluding undetected species, we found that at the phylum level (Figure 6D), only Bacillota showed a significant positive correlation with butyrate, villus length, crypt depth, and muscular layer thickness (*p* < 0.05). In contrast, Planctomycetota, Acidobacteriota, and Myxococcota exhibited significant negative correlations with certain fermentation parameters and tissue morphology indices (*p* < 0.05). At the genus level (Figure 6E), *Clostridium* and *Tepidibacter* displayed significant positive correlations with villus length, muscular layer thickness, and crypt depth (*p* < 0.05). *Paenibacillus* demonstrated a highly significant positive correlation with pH, acetate, propionate, and TVFAs (*p* < 0.01), along with a significant positive correlation with crypt depth (*p* < 0.05). *Mageeibacillus* showed an extremely significant positive correlation with butyrate, crypt depth, villus length, and muscular layer thickness (*p* < 0.01), as well as a highly significant positive correlation with pH (*p* < 0.01) and significant positive correlations with propionate and butyrate (*p* < 0.05). *Kandleria*, *Rhodococcus*, and *Phocaeicola* exhibited significant negative correlations with pH, propionate, butyrate, and villus length (*p* < 0.05) or extremely significant negative correlations (*p* < 0.01). These findings indicate that Bacillota, *Clostridium, Tepidibacter, Paenibacillus*, *Mageeibacillus*, and *Ercella* demonstrate strong positive correlations with fermentation parameters and various gut tissue morphology indices.

#### 3.6.2 Prediction of gut microbial gene function and correlation analysis between microbial community and fermentation parameters

**FIGURE 7:**
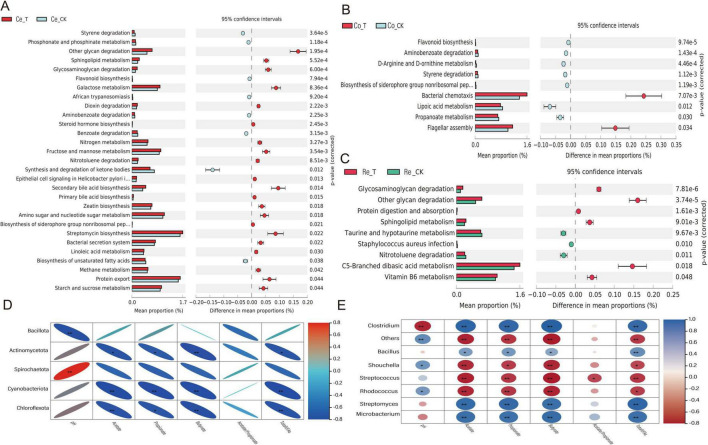
KEGG pathway predictions and interaction correlation heatmaps. **(A)** KEGG functional prediction analysis between cecal microbes. **(B)** KEGG functional prediction analysis between colonic microbes. **(C)** KEGG functional prediction analysis between rectal microbes. **(D)** Correlation heatmap between colon microbiota and fermentation parameters (top 20 genera with the highest relative abundance). **(E)** Correlation heatmap between colon microbiota and fermentation parameters (top 20 phyla with the highest relative abundance). Adjusted *p < 0.05 and **p < 0.01 by Student’s *t*-test.

As described in section 2.6.1, the microbial gene sequences were functionally predicted using PICRUSt 2 software, resulting in the identification of 47 KEGG gene families in the colon. Among these, 29 pathways exhibited significant differences (*p* < 0.05) between the T group and the CK group in the cecum. Specifically, 19 pathways, including other glycan degradation, Glycosaminoglycan degradation, Galactose metabolism, Secondary bile acid biosynthesis, Amino sugar and nucleotide sugar metabolism, Streptomycin biosynthesis, Protein export, and Starch and sucrose metabolism, demonstrated higher proportions in the T group (Figure 7A). In both the colon and rectum, 9 pathways each showed significant differences (*p* < 0.05). In the colon, two pathways, Bacterial chemotaxis and Flagellar assembly, exhibited higher proportions in the T group compared to the CK group (Figure 7B). In the rectum, six pathways, including other glycan degradation, Glycosaminoglycan degradation, Protein digestion and absorption, Sphingolipid metabolism, C5-Branched dibasic acid metabolism, and Vitamin B6 metabolism, displayed higher proportions in the T group (Figure 7C). These results suggest that CSF may play significant roles in carbohydrate metabolism, lipid and nitrogen metabolism, energy metabolism, immune regulation, and bacterial motility mechanisms within the colon microbiota of sheep.

Spearman correlation analysis indicated that, at the phylum level (Figure 7D), Spirochaetota exhibited a significant positive correlation with pH (*p* < 0.01). Conversely, Actinomycetota, Cyanobacteriota, and Chloroflexota demonstrated significant negative correlations with various fermentation parameters (*p* < 0.05). At the genus level (Figure 7E), *Clostridium, Bacillus, Streptomyces*, and *Microbacterium* were significantly positively correlated with Acetate, Propionate, Butyrate, and TVFAs (*p* < 0.01); Additionally, *Schouchella* and *Rhodococcus* showed a significant positive correlation with pH but exhibited significant negative correlations with Acetate, Propionate, and other indices (*p* < 0.05). Meanwhile, *Streptococcus* displayed significant negative correlations with fermentation parameter indices (*p* < 0.01). Overall, Spirochaetota, *Clostridium*, *Bacillus, Streptomyces* and *Microbacterium* were found to have strong positive correlations with fermentation parameters.

## 4 Discussion

### 4.1 Effects of Chive seed flavonoid on intestinal fermentation parameters and histomorphology in sheep

The intestine is a critical site for nutrient absorption, metabolic regulation, and further digestion in ruminants. Among factors influencing intestinal health, pH serves as a key indicator of internal environmental stability, reflecting microbial activity and digestive efficiency. In the present study, dietary supplementation with 0.25% CSF reduced duodenal and jejunal pH but elevated pH in the ileum and colon. These site-specific pH shifts may stem from CSF’s modulation of gut microbiota composition and fermentation patterns. Notably, VFAs—primarily acetate, propionate, and butyrate—are central microbial metabolites in ruminants, providing 70–80% of the host’s metabolic energy (Ma et al., 2021). Beyond their energetic role, VFAs enhance nutrient absorption (lipids and proteins) in the small intestine and serve as critical substrates for epithelial cells in the ileum and colon (Dalile et al., 2019; Yao et al., 2022). The results of this study indicate that the addition of 0.25% CSF to the diet increased the concentrations of acetate, propionate, butyrate, and TVFAs in the ileum, cecum, colon, and rectum. Previous studies pointed out that flavonoid supplementation can enhance gastrointestinal fermentation parameters and alleviate symptoms of ruminal acidosis (Balcells et al., 2012). Furthermore, *in vitro* fermentation studies have demonstrated that certain flavonoids can increase the levels of ammonia-nitrogen and SCFAs in the gut (Pan et al., 2023). This is similar to the results of previous studies. Furthermore, it also increased propionate and total VFAs in the jejunum. The supplementation with flavonoids (luteolin and quercetin) has been shown to enhance TVFAs production in sheep fecal microbiota (Pan et al., 2023), suggesting that the observed elevation of intestinal TVFAs levels in ruminants may be attributed to specific bioactive constituents within these phytochemical compounds.

Intestinal histomorphology is a critical metric for assessing gut health and function, with key indicators including villus length, crypt depth, muscle layer thickness, and the V/C ratio (Liao and Nyachoti, 2017). Increased villus length expands the intestinal epithelium’s absorptive surface area, directly enhancing nutrient uptake (Casas et al., 2020; Munoz et al., 2023). Conversely, reduced crypt depth reflects accelerated epithelial cell maturation, which improves nutrient absorption efficiency (Rubin and Levin, 2016). Muscle layer thickness correlates with intestinal contractile strength, facilitating nutrient transport, while a higher V/C ratio signifies structural integrity of the mucosa and improved digestive capacity. In this study, the addition of 0.25% CSF to the diet improved villus length, muscle layer thickness, and V/C ratio in the jejunum and ileum, while reducing crypt depth in the small intestine. These findings align with reports that dietary fiber enhances ileal V/C ratios in weaned piglets (Ellner et al., 2021) and that fatty acids modulate jejunal morphology in poultry (Palomar et al., 2023), suggesting CSF may act through analogous nutrient-driven pathways. Quantitative functional gene analysis revealed upregulated expression levels of *IGF1*, *CD81*, *CTNNBL1*, *MGST1*, *GPX*4, and *SLC39A8* in the ileum and cecum epithelium of T group. *IGF1* was observed to suppress cellular autophagy and enhance myocyte proliferation via the mTOR and FoxO signaling pathways (Weeks et al., 2017), suggesting potential associations between increased small intestinal muscularis thickness and *IGF1*-mediated regulation. These findings imply that CSF may improve intestinal morphogenesis through enhanced epithelial gene expression and elevated fatty acid content, potentially potentiating the absorptive and transport capacities of enterocytes. However, systematic investigations are required to elucidate the precise mechanisms underlying these phenotypic improvements and their nutritional implications.

### 4.2 Effects of Chive seed flavonoid on the whole intestinal microbiota of sheep

Full-length bacterial 16S rRNA sequencing, performed using the PacBio Revio platform, is a third-generation sequencing method that enables high-resolution analysis of microbiome diversity by capturing near-complete rRNA gene sequences (Wagner et al., 2016). This approach improves taxonomic annotation accuracy and offers deeper insights into microbial community dynamics. Host-microbe interactions, often symbiotic, are mediated by microbial metabolites that regulate host physiology (Liu et al., 2020; Ren et al., 2018). Alpha diversity, measured through statistical indices such as Shannon and Chao1, quantifies species richness and evenness within a community. Elevated gut microbial alpha diversity is strongly associated with intestinal homeostasis and systemic health, as it enhances resistance to pathogen colonization, supports immune function, and optimizes nutrient metabolism (Li D. et al., 2023). In this study, dietary supplementation with CSF showed a trend toward increased alpha diversity indices, suggesting a potential improvement in microbial community resilience. Beta diversity analyses (PCoA and NMDS) further revealed distinct clustering of gut microbiota between the CSF-treated (T) and control (CK) groups, indicating significant compositional differences. In most mammals, the gut microbiota is dominated by the phyla Firmicutes (recently reclassified as Bacillota) and Bacteroidetes, which collectively comprise over 98% of the community (Benson et al., 2010; Ley et al., 2008; Ley et al., 2006). However, in sheep from this study, the dominant phyla were Bacillota (formerly Firmicutes), Actinobacteriota, and Bacteroidota, with Bacillota being the most abundant. This divergence may reflect species-specific adaptations, age-related shifts, or environmental influences. LEfSe analysis identified significant CSF-induced alterations in microbial abundance, with the T group harboring a greater number of differential biomarkers than the CK group. Notably, Cyanobacteria (phylum level) and *Bacillus* (genus level) were enriched in the T group. While Cyanobacteria are atypical in gut microbiomes, their presence could indicate dietary influence or ecological niche specialization. The prominence of *Bacillus*, a genus known for metabolic versatility, aligns with CSF’s potential to modulate nutrient-processing taxa. These structural shifts suggest CSF enhances gut microbiota functionality, possibly synergizing with its previously reported effects on volatile fatty acid production and intestinal morphogenesis.

#### 4.2.1 Effect of Chive seed flavonoid on the intestinal microbiota of sheep small intestine

The small intestine, particularly the jejunum and ileum, plays a pivotal role in nutrient absorption and digestion. As the primary site for nutrient uptake, a healthy jejunum is critical for animal growth and overall health. The ileum, positioned downstream of the jejunum, further contributes to digestion and absorption processes (Godoy-Vitorino et al., 2012). To evaluate the impact of CSF on gut microbiota, the study analyzed microbial communities across intestinal segments. This study results revealed distinct compositional differences between the large and small intestines. At the phylum level, Firmicutes and Bacteroidetes were identified as the most abundant microbial communities in the small intestine of sheep. The abundance of Clostridia increased significantly in the ileum, which aligns with its beneficial effects, such as inhibiting the proliferation of harmful bacteria, promoting the growth of beneficial bacteria, enhancing villus height and the V/C ratio, restoring intestinal permeability, and improving nutrient absorption. The observed improvements in ileal villus length, muscle layer thickness, and V/C ratio in this study can be attributed to the promotion of Clostridia abundance by CSF, which, in turn, fosters intestinal development. Additionally, the abundance of Bacteroidetes increased significantly in the duodenum, consistent with previous reports indicating that the duodenum and jejunum are primarily composed of Firmicutes and Bacteroidetes (Di Rienzi et al., 2013), Firmicutes and Bacteroidetes, the most diverse microbial communities in nature, exhibit exceptional metabolic capabilities and play a key role in immune regulation, particularly in the production of antibiotics (Barka et al., 2016; van Bergeijk et al., 2020). Bacteroides and Cyanobacteria exhibited significant increases throughout the small intestine. Previous studies have demonstrated that Bacteroides are particularly effective at degrading carbohydrates and polysaccharides, metabolizing these compounds into VFAs (Fan et al., 2024). Cyanobacteria, as key components of the sheep gut microbiome, perform a variety of functions, including the synthesis of vitamin B, anaerobic fermentation, oxygen-dependent hydrogen production, nitrogen fixation, and the degradation of hemicellulose and pectin, as well as methane reduction (Huo et al., 2014). At the genus level, *Clostridium* (phylum Firmicutes) was highly abundant in the small intestine, with increased levels in the jejunum and ileum. This genus enhances host energy absorption and immune regulation (Reuss et al., 2016) and produces butyrate, a short-chain fatty acid critical for gut health and diarrhea prevention (Benson et al., 2010). Similarly, *Streptomyces* (phylum Actinobacteria) surged in the ileum. Known for metabolic diversity, *Streptomyces* synthesizes bioactive metabolites that strengthen the intestinal barrier by blocking pathogens (Odchimar et al., 2024). Both genera degrade dietary fiber into VFAs, aligning with elevated VFA levels in CSF-supplemented sheep. LEfSe analysis revealed that the small intestinal microbial community enriched by CSF in sheep belonged to the phyla Firmicutes, Bacteroidetes, Bacteroides, and Clostridia, with *Lachnospiraceae* and *Micrococcales* (both belonging to Firmicutes) serving as specific biomarkers for the jejunum. These bacteria are potentially beneficial, as *Lachnospiraceae* are involved in the metabolism of various carbohydrates (Zhang et al., 2023) and *Micrococcales* contribute to metabolic activities in the intestine, thereby maintaining the stability of the sheep gut barrier. Functional pathway enrichment analysis indicated that the number of microbial gene pathways enriched in the small intestine was highest in the ileum, followed by the jejunum and duodenum. These pathways are implicated in multiple physiological and biochemical processes, with significantly enriched pathways in the treatment group primarily focusing on protein synthesis and processing, immune responses, amino acid metabolism, signal transduction, energy metabolism, and the transmission and expression of genetic information (e.g., glycan degradation, fatty acid biosynthesis, protein processing in the endoplasmic reticulum, and mRNA surveillance pathways). These pathways correlate with upregulated genes (*IGF1*, *CD81*, *CTNNBL1*), which enhance antioxidant and immune functions (Shahsavari et al., 2023; Xiao et al., 2021), suggesting CSF boosts immunocompetence via microbiota-host crosstalk. The ileum’s role in the gut-liver axis and amino acid absorption underscores its importance in nutrient metabolism, consistent with CSF’s effects on ileal morphology and VFA production (Liu et al., 2024). Additionally, gut microbiota may amplify CSF’s benefits by metabolizing flavonoids (Li Z. et al., 2023). Further emphasizing the essential role of the ileum in nutrient digestion and absorption in the small intestine, consistent with our findings.

Correlation heatmap analysis revealed Firmicutes (phylum) positively correlated with butyrate levels, villus length, crypt depth, and muscle layer thickness. This aligns with its known role in enhancing intestinal structural integrity (Fan et al., 2024). In contrast, Bacteroidetes and Actinobacteria showed negative correlations with villus length and crypt depth. While Actinobacteria maintain mucosal barriers via antibiotic production and Bacteroidetes regulate polysaccharide uptake and metabolic health (White et al., 2023), their limited direct impact on mucosal growth may explain these inverse trends. At the genus level, Clostridium, *Tepidibacter*, *Mageeibacillus*, *Paenibacillus*, and *Ercella* correlated positively with fermentation metrics and histomorphology. *Clostridium*, a beneficial symbiont, modulates microbiota, produces nutrients, and enhances immunity (Guo et al., 2020). *Mageeibacillus* (thermophilic fermenter) and *Paenibacillus* degrade carbohydrates/proteins into metabolites like acetate, while *Ercella* produces SCFAs for barrier integrity. Notably, *Paenibacillus* may reduce methane by competing with methanogens (Justine et al., 2025), Although *Mageeibacillus*’s role in butyrate synthesis and mucosal development remains understudied (Austin et al., 2015), its correlation with histomorphological improvements highlights its potential as a probiotic target.

#### 4.2.2 Effect of Chive seed flavonoid on the intestinal microbiota of sheep large intestine

The large intestine, particularly the cecum, is a key site for microbial fermentation in herbivores, hosting diverse microbiota critical for digestion and health (Ballout and Diener, 2021). Unlike the rumen, the cecum specializes in breaking down recalcitrant polymers like lignin and starch (Godoy-Vitorino et al., 2012). Cecal fermentation produces 12% of TVFAs and contributes 17% of cellulose digestion (Dixon and Nolan, 1982), aligning with our finding of elevated VFAs levels in the cecum. Downstream, the colon houses carbohydrate-degrading microbiota that supply energy (Lin et al., 2021) and modulate immune-linked diseases (O’Keefe, 2016; Yan et al., 2017). The rectum, the terminal segment, ensures waste expulsion and homeostasis by preventing toxin accumulation. Microbial richness and diversity were higher in the large intestine than the small intestine. Bacillota (formerly Firmicutes) and Bacteroidota (formerly Bacteroidetes) dominated, with CSF increasing Actinobacteriota abundance in the cecum and colon. Actinobacteriota regulates gut permeability, immunity, and the gut-brain axis, offering therapeutic potential (Binda et al., 2018). Accumulating evidence indicates that dietary flavonoids suppress inflammatory cascades, modulate immune cell functionality, and preserve intestinal barrier integrity through microbiota-dependent pathways (Wang et al., 2024). Notably, CSF elevated Bacteroidota in the rectum, possibly stabilizing gut metabolism, though reduced Bacteroidota in other segments suggests niche-specific ecological shifts (Lin et al., 2021). These findings suggest that CSF enhances Bacteroidota abundance to stabilize gut health-related metabolic processes. At the genus level, *Clostridium* was the most abundant bacterium in the large intestine, though CSF minimally affected its abundance. Increased *Clostridium* in the small intestine may stem from environmental variations there. *Blautia* and *Bacteroides* were less abundant than *Clostridium*, consistent with prior studies (Ye et al., 2022). *Bacteroides* aids complex carbohydrate digestion (Spence et al., 2006), while *Blautia* influences metabolism and inflammation (Liu et al., 2021). Additionally, *Shouchella* exhibited increased abundance in the cecum and colon, suggesting its potential as a beneficial probiotic with protective gut functions. LEfSe analysis showed CSF shifted cecal dominance to Bacillaceae (phylum Bacillota), key for nutrient degradation (Gebeyew et al., 2021). In the colon, discriminatory taxa included *Sphingobacteriia* (class), *Sphingobacteriales* (order), and *Sphingobacteriaceae* (family) (phylum Bacteroidetes), reflecting microbiota instability (Smoliński et al., 2021). Rectal biomarkers aligned with Bacteroidetes dominance (Zhang et al., 2018). KEGG analysis highlighted cecal dominance in pathways like energy, lipid, and toxin metabolism. CSF enriched cecal genes in carbohydrate/galactose metabolism and protein turnover—critical for growth and adaptation (González and Cullen, 2022). Lipid metabolism dysregulation links to disease (Petrenko et al., 2023). In the colon, pathways such as bacterial chemotaxis and flagellar assembly exhibited significantly increased microbial gene enrichment. Bacterial chemotaxis is a strategy employed by bacteria to enhance nutrient and energy uptake (Keegstra et al., 2022), while bacterial flagella are large protein machines essential for motility and biofilm formation, processes critical for bacterial survival and pathogenesis (Takekawa et al., 2020). In the rectum, pathways associated with glycan metabolism, vitamin B6 metabolism, and protein digestion exhibited significantly increased microbial gene enrichment. Crucially, intestinal microorganisms engage in mutualistic symbiosis with flavonoid compounds, wherein microbial metabolism enhances flavonoid bioactivity while these phytochemicals reciprocally shape microbial community structure to sustain gut homeostasis (Williamson et al., 2018), supporting CSF as a phytogenic additive with microbiota-modulating potential.

Correlation analysis between microbiota and fermentation parameters revealed that Spiraeochnusella is positively correlated with pH, playing a significant role in regulating gut microecological balance and serving as a primary degrader of lignocellulose (Lin et al., 2018). Genera such as *Clostridium, Bacillus, Streptococcus* and *Microbacterium* showed positive correlations with acetate, propionate, butyrate, and TVFAs. *Clostridiu*, a member of Firmicutes, facilitates the degradation of fibers and polysaccharides, thereby promoting growth and development. *Bacillus* and *Streptococcus* can utilize sugar-based substrates and exhibit strong hydrolytic capacities for starch and proteins (Sánchez-Moya et al., 2017), while *Microbacterium*, an aerobic bacterium, likely possesses the ability to degrade complex carbohydrates and enhance nutrient absorption and utilization based on the correlation heatmap findings.

In summary, CSF enhances the abundance of beneficial gut bacteria, thereby promoting effective fermentation and utilization of feed, as well as carbohydrate and lipid metabolism, immune regulation, and the maintenance of gut barrier stability through multiple mechanisms. These effects positively influence the health and growth of sheep.

## 5 Conclusion

Dietary supplementation with 0.25% CSF enhanced intestinal development and microbiota in sheep. CSF increased jejunal and ileal villus length, V/C ratio, and muscle layer thickness, while reducing crypt depth. It elevated acetate, propionate, butyrate, and TVFAs in the ileum, cecum, colon, and rectum, and boosted propionate and TVFAs in the jejunum. Notably, the addition of CSF significantly optimized the structure of beneficial bacteria in the gut. T group also upregulated the expression of ileum and cecum epithelial genes (*IGF1*, *CD81*, *CTNNBL1*, *MGST1*, *GPX4*, *SLC39A8*) associated with the regulation of tissue morphogenesis and VFAs absorption. Specifically, the abundances of Bacteroidota and Cyanobacteriota were elevated throughout the small intestine, while Actinobacteria abundance increased in the duodenum. In the jejunum, the abundance of Clostridia increased, and in the ileum, the abundances of both *Clostridium* and *Streptomyces* were elevated. Additionally, Actinobacteria abundances were increased in both the cecum and colon, and Bacteroidota abundance rose in the rectum. Functional predictions suggested that CSF may promote carbohydrate metabolism, energy metabolism, immune regulation, and maintenance of the intestinal barrier. Therefore, these findings suggest that CSF may enhance VFAs biosynthesis by modulating the composition and metabolic functions of the intestinal microbiota in sheep. Concurrently, VFAs serve as critical energy substrates for intestinal epithelial cells, activating gene expression associated with tissue morphogenesis and development. This process drives intestinal villi hyperplasia and crypt structure maturation, thereby improving VFAs absorption capacity and establishing a synergistic regulatory mechanism of “microbial metabolism-morphological development-nutrient absorption.” This evidence confirms the feasibility of utilizing CSF as botanical feed additives, providing a theoretical foundation for advancing nutritional regulation strategies in ruminant husbandry.

## Data Availability

The original contributions presented in the study are publicly available. This data can be found at: https://www.ncbi.nlm.nih.gov/, accession number: PRJNA1225640.

## References

[B1] Amanzadeh E. Esmaeili A. Rahgozar S. Nourbakhshnia M. (2019). Application of quercetin in neurological disorders: From nutrition to nanomedicine. *Rev. Neurosci.* 30 555–572. 10.1515/revneuro-2018-0080 30753166

[B2] Austin M. N. Rabe L. K. Srinivasan S. Fredricks D. N. Wiesenfeld H. C. Hillier S. L. (2015). *Mageeibacillus indolicus* gen. nov., sp. nov.: A novel bacterium isolated from the female genital tract. *Anaerobe* 32 37–42. 10.1016/j.anaerobe.2014.12.003 25482717 PMC4385425

[B3] Balcells J. Aris A. Serrano A. Seradj A. R. Crespo J. Devant M. (2012). Effects of an extract of plant flavonoids (Bioflavex) on rumen fermentation and performance in heifers fed high-concentrate diets. *J. Anim. Sci.* 90 4975–4984. 10.2527/jas.2011-4955 22829622

[B4] Ballout J. Diener M. (2021). The role of HCO(3)(-) in propionate-induced anion secretion across rat caecal epithelium. *Pflugers Arch.* 473 937–951. 10.1007/s00424-021-02565-8 33914143 PMC8164622

[B5] Barka E. A. Vatsa P. Sanchez L. Gaveau-Vaillant N. Jacquard C. Meier-Kolthoff J. P. (2016). Taxonomy, physiology, and natural products of actinobacteria. *Microbiol. Mol. Biol. Rev.* 80 1–43. 10.1128/mmbr.00019-15 26609051 PMC4711186

[B6] Benson A. K. Kelly S. A. Legge R. Ma F. Low S. J. Kim J. (2010). Individuality in gut microbiota composition is a complex polygenic trait shaped by multiple environmental and host genetic factors. *Proc. Natl. Acad. Sci. U.S.A.* 107 18933–18938. 10.1073/pnas.1007028107 20937875 PMC2973891

[B7] Binda C. Lopetuso L. R. Rizzatti G. Gibiino G. Cennamo V. Gasbarrini A. (2018). Actinobacteria: A relevant minority for the maintenance of gut homeostasis. *Dig. Liver Dis.* 50 421–428. 10.1016/j.dld.2018.02.012 29567414

[B8] Bolyen E. Rideout J. R. Dillon M. R. Bokulich N. A. Abnet C. C. Al-Ghalith G. A. (2019). Author Correction: Reproducible, interactive, scalable and extensible microbiome data science using QIIME 2. *Nat. Biotechnol.* 37:1091. 10.1038/s41587-019-0252-6 31341288 PMC7015180

[B9] Buiatte V. Proszkowiec-Weglarz M. Miska K. Dominguez D. Mahmoud M. Lesko T. (2024). The effects of a high-flavonoid corn cultivar on the gastrointestinal tract microbiota in chickens undergoing necrotic enteritis. *PLoS One* 19:e0307333. 10.1371/journal.pone.0307333 39288108 PMC11407631

[B10] Casas G. A. Blavi L. Cross T. L. Lee A. H. Swanson K. S. Stein H. H. (2020). Inclusion of the direct-fed microbial Clostridium butyricum in diets for weanling pigs increases growth performance and tends to increase villus height and crypt depth, but does not change intestinal microbial abundance. *J. Anim. Sci.* 98:skz372. 10.1093/jas/skz372 31820779 PMC6986446

[B11] Chen D. Chen X. Tu Y. Wang B. Lou C. Ma T. (2015). Effects of mulberry leaf flavonoid and resveratrol on methane emission and nutrient digestion in sheep. *Anim. Nutr.* 1 362–367. 10.1016/j.aninu.2015.12.008 29767046 PMC5940990

[B12] Dalile B. Van Oudenhove L. Vervliet B. Verbeke K. (2019). The role of short-chain fatty acids in microbiota-gut-brain communication. *Nat. Rev. Gastroenterol. Hepatol.* 16 461–478. 10.1038/s41575-019-0157-3 31123355

[B13] de Freitas K. S. Squarisi I. S. Acésio N. O. Nicolella H. D. Ozelin S. D. Reis Santos (2020). Licochalcone A, a licorice flavonoid: antioxidant, cytotoxic, genotoxic, and chemopreventive potential. *J. Toxicol. Environ. Health A* 83 673–686. 10.1080/15287394.2020.1813228 32886024

[B14] Di Rienzi S. C. Sharon I. Wrighton K. C. Koren O. Hug L. A. Thomas B. C. (2013). The human gut and groundwater harbor non-photosynthetic bacteria belonging to a new candidate phylum sibling to Cyanobacteria. *Elife* 2:e01102. 10.7554/eLife.01102 24137540 PMC3787301

[B15] Dixon R. M. Nolan J. V. (1982). Studies of the large intestine of sheep. 1. Fermentation and absorption in sections of the large intestine. *Br. J. Nutr.* 47 289–300. 10.1079/bjn19820038 7066290

[B16] Ellner C. Martínez-Vallespín B. Saliu E. M. Zentek J. Röhe I. (2021). Effects of cereal and protein source on performance, apparent ileal protein digestibility and intestinal characteristics in weaner piglets. *Arch. Anim. Nutr.* 75 263–277. 10.1080/1745039x.2021.1958647 34427485

[B17] Fan D. Fu Y. Zhang J. Bi Y. Ma T. Diao Q. (2024). Sheep-derived butyrate-producing *Clostridium beijerinckii* R8 alleviates diarrhea by shaping the gut microbiota of goat kids. *Anim. Nutr.* 19 13–24. 10.1016/j.aninu.2024.06.004 39624508 PMC11609465

[B18] Gebeyew K. Chen K. Wassie T. Azad M. A. K. He J. Jiang W. (2021). Dietary amylose/amylopectin ratio modulates cecal microbiota and metabolites in weaned goats. *Front. Nutr.* 8:774766. 10.3389/fnut.2021.774766 34957184 PMC8697430

[B19] Godoy-Vitorino F. Goldfarb K. C. Karaoz U. Leal S. Garcia-Amado M. A. Hugenholtz P. (2012). Comparative analyses of foregut and hindgut bacterial communities in hoatzins and cows. *ISME J.* 6 531–541. 10.1038/ismej.2011.131 21938024 PMC3280141

[B20] González B. Cullen P. J. (2022). Regulation of Cdc42 protein turnover modulates the filamentous growth MAPK pathway. *J. Cell Biol.* 221:e202112100. 10.1083/jcb.202112100 36350310 PMC9811999

[B21] Guo P. Zhang K. Ma X. He P. (2020). Clostridium species as probiotics: Potentials and challenges. *J. Anim. Sci. Biotechnol.* 11:24. 10.1186/s40104-019-0402-1 32099648 PMC7031906

[B22] Hooper L. V. Macpherson A. J. (2010). Immune adaptations that maintain homeostasis with the intestinal microbiota. *Nat. Rev. Immunol.* 10 159–169. 10.1038/nri2710 20182457

[B23] Huo W. Zhu W. Mao S. (2014). Impact of subacute ruminal acidosis on the diversity of liquid and solid-associated bacteria in the rumen of goats. *World J. Microbiol. Biotechnol.* 30 669–680. 10.1007/s11274-013-1489-8 24068532

[B24] Justine E. E. Lee H. J. Jung K. H. Lee Y. S. Kim Y. J. (2025). Methane emission mitigation of *Paenibacillus yonginensis* DCY84(T) incorporated with silicate on paddy rice (*Oryzae sativa* L.) plantation revealed in soil microbiome profiling. *Sci. Total Environ.* 958:177996. 10.1016/j.scitotenv.2024.177996 39671945

[B25] Keegstra J. M. Carrara F. Stocker R. (2022). The ecological roles of bacterial chemotaxis. *Nat. Rev. Microbiol.* 20 491–504. 10.1038/s41579-022-00709-w 35292761

[B26] Lee W. J. Hase K. (2014). Gut microbiota-generated metabolites in animal health and disease. *Nat. Chem. Biol.* 10 416–424. 10.1038/nchembio.1535 24838170

[B27] Ley R. E. Hamady M. Lozupone C. Turnbaugh P. J. Ramey R. R. Bircher J. S. (2008). Evolution of mammals and their gut microbes. *Science* 320 1647–1651. 10.1126/science.1155725 18497261 PMC2649005

[B28] Ley R. E. Turnbaugh P. J. Klein S. Gordon J. I. (2006). Microbial ecology: Human gut microbes associated with obesity. *Nature* 444 1022–1023. 10.1038/4441022a 17183309

[B29] Li D. Yang H. Li Q. Ma K. Wang H. Wang C. (2023). Prickly Ash Seeds improve immunity of Hu sheep by changing the diversity and structure of gut microbiota. *Front. Microbiol.* 14:1273714. 10.3389/fmicb.2023.1273714 38029081 PMC10644117

[B30] Li Q. Wu Y. Qi X. Liu Z. Wang C. Ma X. (2024). Effects of prickly ash seed dietary supplementation on meat quality, antioxidative capability, and metabolite characteristics of hu lambs. *Foods* 13:3415. 10.3390/foods13213415 39517199 PMC11545103

[B31] Li Z. Ren Z. Zhao L. Chen L. Yu Y. Wang D. (2023). Unique roles in health promotion of dietary flavonoids through gut microbiota regulation: Current understanding and future perspectives. *Food Chem.* 399:133959. 10.1016/j.foodchem.2022.133959 36001928

[B32] Liao S. F. Nyachoti M. (2017). Using probiotics to improve swine gut health and nutrient utilization. *Anim. Nutr.* 3 331–343. 10.1016/j.aninu.2017.06.007 29767089 PMC5941265

[B33] Lin L. Trabi E. B. Xie F. Mao S. (2021). Comparison of the fermentation and bacterial community in the colon of Hu sheep fed a low-grain, non-pelleted, or pelleted high-grain diet. *Appl. Microbiol. Biotechnol.* 105 2071–2080. 10.1007/s00253-021-11158-5 33559720

[B34] Lin X. Wang J. Hou Q. Wang Y. Hu Z. Shi K. (2018). Effect of hay supplementation timing on rumen microbiota in suckling calves. *Microbiologyopen* 7:e00430. 10.1002/mbo3.430 29280327 PMC5822350

[B35] Liu X. Mao B. Gu J. Wu J. Cui S. Wang G. (2021). Blautia-a new functional genus with potential probiotic properties? *Gut Microbes* 13 1–21. 10.1080/19490976.2021.1875796 33525961 PMC7872077

[B36] Liu X. Sha Y. Dingkao R. Zhang W. Lv W. Wei H. (2020). Interactions between rumen microbes, VFAs, and host genes regulate nutrient absorption and epithelial barrier function during cold season nutritional stress in tibetan sheep. *Front. Microbiol.* 11:593062. 10.3389/fmicb.2020.593062 33250882 PMC7674685

[B37] Liu Z. Wang H. Ma K. Li Q. Wu Y. Qi X. (2024). Supplementation with Chinese herbal preparations protect the gut-liver axis of Hu sheep, promotes gut-liver circulation, regulates intestinal flora and immunity. *Front. Immunol.* 15:1454334. 10.3389/fimmu.2024.1454334 39606237 PMC11599181

[B38] Ma J. Wang C. Wang Z. Cao G. Hu R. Wang X. (2021). Active dry yeast supplementation improves the growth performance, rumen fermentation, and immune response of weaned beef calves. *Anim. Nutr.* 7 1352–1359. 10.1016/j.aninu.2021.06.006 34786508 PMC8577086

[B39] Munoz L. R. Bailey M. A. Krehling J. T. Bourassa D. V. Hauck R. Pacheco W. J. (2023). Effects of dietary yeast cell wall supplementation on growth performance, intestinal *Campylobacter jejuni* colonization, innate immune response, villus height, crypt depth, and slaughter characteristics of broiler chickens inoculated with *Campylobacter jejuni* at d 21. *Poult. Sci.* 102:102609. 10.1016/j.psj.2023.102609 36963334 PMC10060741

[B40] Muqier, Qi S. Wang T. Chen R. Wang C. Ao C. (2017). Effects of flavonoids from *Allium mongolicum* Regel on growth performance and growth-related hormones in meat sheep. *Anim. Nutr.* 3 33–38. 10.1016/j.aninu.2017.01.003 29767126 PMC5941079

[B41] Nakai S. Kamei Y. (2020). Health promotion effects of soy isoflavones. *J. Nutr. Sci. Vitaminol. (Tokyo)* 66 502–507. 10.3177/jnsv.66.502 33390391

[B42] Odchimar N. M. O. Macalalad M. A. B. Orosco F. L. (2024). From antibiotic to antiviral: Computational screening reveals a multi-targeting antibiotic from *Streptomyces* spp. against Nipah virus fusion proteins. *Mol. Divers.* 29 1541–1555. 10.1007/s11030-024-10932-7 39060858

[B43] O’Keefe S. J. (2016). Diet, microorganisms and their metabolites, and colon cancer. *Nat. Rev. Gastroenterol. Hepatol.* 13 691–706. 10.1038/nrgastro.2016.165 27848961 PMC6312102

[B44] Oteiza P. I. Fraga C. G. Mills D. A. Taft D. H. (2018). Flavonoids and the gastrointestinal tract: Local and systemic effects. *Mol. Aspects Med.* 61 41–49. 10.1016/j.mam.2018.01.001 29317252

[B45] Palomar M. Garcés-Narro C. Piquer O. Sala R. Tres A. García-Bautista J. A. (2023). Influence of free fatty acid content and degree of fat saturation on production performance, nutrient digestibility, and intestinal morphology of laying hens. *Anim. Nutr.* 13 313–323. 10.1016/j.aninu.2023.03.002 37197305 PMC10184043

[B46] Pan L. Ye H. Pi X. Liu W. Wang Z. Zhang Y. (2023). Effects of several flavonoids on human gut microbiota and its metabolism by in vitro simulated fermentation. *Front. Microbiol.* 14:1092729. 10.3389/fmicb.2023.1092729 36819019 PMC9932666

[B47] Petrenko V. Sinturel F. Riezman H. Dibner C. (2023). Lipid metabolism around the body clocks. *Prog. Lipid Res.* 91:101235. 10.1016/j.plipres.2023.101235 37187314

[B48] Ren W. Wang P. Yan J. Liu G. Zeng B. Hussain T. (2018). Melatonin alleviates weanling stress in mice: Involvement of intestinal microbiota. *J. Pineal Res.* 64:e12448. 10.1111/jpi.12448 28875556

[B49] Reuss B. Asif A. R. Almamy A. Schwerk C. Schroten H. Ishikawa H. (2016). Antisera against *Neisseria gonorrhoeae* cross-react with specific brain proteins of the common marmoset monkey and other nonhuman primate species. *Brain Res.* 1653 23–38. 10.1016/j.brainres.2016.10.012 27765579

[B50] Rubin D. C. Levin M. S. (2016). Mechanisms of intestinal adaptation. *Best Pract. Res. Clin. Gastroenterol.* 30 237–248. 10.1016/j.bpg.2016.03.007 27086888 PMC4874810

[B51] Sánchez-Moya T. López-Nicolás R. Planes D. González-Bermúdez C. A. Ros-Berruezo G. Frontela-Saseta C. (2017). In vitro modulation of gut microbiota by whey protein to preserve intestinal health. *Food Funct.* 8 3053–3063. 10.1039/c7fo00197e 28636003

[B52] Sang S. M. Xia Z. H. Mao S. L. Lao A. N. Chen Z. L. (2000). [Studies on chemical constitutents in seeds of *Allium tuberosum* Rottl]. *Zhongguo Zhong Yao Za Zhi* 25 286–288. 10.1186/s12906-019-2542-4 12512450

[B53] Shahsavari M. Mohammadabadi M. Khezri A. Asadi Fozi M. Babenko O. Kalashnyk O. (2023). Correlation between insulin-like growth factor 1 gene expression and fennel (*Foeniculum vulgare*) seed powder consumption in muscle of sheep. *Anim. Biotechnol.* 34 882–892. 10.1080/10495398.2021.2000997 34783639

[B54] Shi J. Lei Y. Wu J. Li Z. Zhang X. Jia L. (2023). Antimicrobial peptides act on the rumen microbiome and metabolome affecting the performance of castrated bulls. *J. Anim. Sci. Biotechnol.* 14:31. 10.1186/s40104-023-00832-5 36890581 PMC9996874

[B55] Smoliński J. Szeligowska N. Cholewińska P. Czyż K. Janczak M. (2021). Levels of main bacterial phyla in the gastrointestinal tract of sheep depending on parity and age. *Animals (Basel)* 11:2203. 10.3390/ani11082203 34438660 PMC8388517

[B56] Spence C. Wells W. G. Smith C. J. (2006). Characterization of the primary starch utilization operon in the obligate anaerobe *Bacteroides fragilis*: Regulation by carbon source and oxygen. *J. Bacteriol.* 188 4663–4672. 10.1128/jb.00125-06 16788175 PMC1482989

[B57] Takekawa N. Imada K. Homma M. (2020). Structure and energy-conversion mechanism of the bacterial Na(+)-driven flagellar motor. *Trends Microbiol.* 28 719–731. 10.1016/j.tim.2020.03.010 32781026

[B58] van Bergeijk D. A. Terlouw B. R. Medema M. H. van Wezel G. P. (2020). Ecology and genomics of Actinobacteria: New concepts for natural product discovery. *Nat. Rev. Microbiol.* 18 546–558. 10.1038/s41579-020-0379-y 32483324

[B59] van Dijk A. Hedegaard C. J. Haagsman H. P. Heegaard P. M. H. (2018). The potential for immunoglobulins and host defense peptides (HDPs) to reduce the use of antibiotics in animal production. *Vet. Res.* 49:68. 10.1186/s13567-018-0558-2 30060758 PMC6066942

[B60] Wagner J. Coupland P. Browne H. P. Lawley T. D. Francis S. C. Parkhill J. (2016). Evaluation of PacBio sequencing for full-length bacterial 16S rRNA gene classification. *BMC Microbiol.* 16:274. 10.1186/s12866-016-0891-4 27842515 PMC5109829

[B61] Wang L. Li M. Gu Y. Shi J. Yan J. Wang X. (2024). Dietary flavonoids-microbiota crosstalk in intestinal inflammation and carcinogenesis. *J. Nutr. Biochem.* 125:109494. 10.1016/j.jnutbio.2023.109494 37866426

[B62] Weeks K. L. Bernardo B. C. Ooi J. Y. Y. Patterson N. L. McMullen J. R. (2017). The IGF1-PI3K-Akt signaling pathway in mediating exercise-induced cardiac hypertrophy and protection. *Adv. Exp. Med. Biol.* 1000 187–210. 10.1007/978-981-10-4304-8_12 29098623

[B63] White J. B. R. Silale A. Feasey M. Heunis T. Zhu Y. Zheng H. (2023). Outer membrane utilisomes mediate glycan uptake in gut Bacteroidetes. *Nature* 618 583–589. 10.1038/s41586-023-06146-w 37286596 PMC7618045

[B64] Williamson G. Kay C. D. Crozier A. (2018). The bioavailability, transport, and bioactivity of dietary flavonoids: A review from a historical perspective. *Compr. Rev. Food Sci. Food Saf.* 17 1054–1112. 10.1111/1541-4337.12351 33350159

[B65] Xiao C. Jin H. G. Zhang L. C. Liu J. Q. He M. Ma H. H. (2021). Effects of SPARCL1 on the proliferation and differentiation of sheep preadipocytes. *Adipocyte* 10 658–669. 10.1080/21623945.2021.2010901 34872433 PMC8654481

[B66] Yan S. Zhu C. Yu T. Huang W. Huang J. Kong Q. (2017). Studying the differences of bacterial metabolome and microbiome in the colon between landrace and meihua piglets. *Front. Microbiol.* 8:1812. 10.3389/fmicb.2017.01812 28983290 PMC5613163

[B67] Yao Y. Cai X. Fei W. Ye Y. Zhao M. Zheng C. (2022). The role of short-chain fatty acids in immunity, inflammation and metabolism. *Crit. Rev Food Sci. Nutr.* 62 1–12. 10.1080/10408398.2020.1854675 33261516

[B68] Ye M. Hou M. Peng Q. Jia S. Peng B. Yin F. (2022). The microbiota and cytokines correlation between the jejunum and colon in altay sheep. *Animals (Basel)* 12:1564. 10.3390/ani12121564 35739900 PMC9219508

[B69] Zhang H. Shao M. Huang H. Wang S. Ma L. Wang H. (2018). The dynamic distribution of small-tail han sheep microbiota across different intestinal segments. *Front Microbiol* 9:32. 10.3389/fmicb.2018.00032 29445360 PMC5797768

[B70] Zhang H. Zha X. Zhang B. Zheng Y. Elsabagh M. Wang H. (2024). Gut microbiota contributes to bisphenol A-induced maternal intestinal and placental apoptosis, oxidative stress, and fetal growth restriction in pregnant ewe model by regulating gut-placental axis. *Microbiome* 12:28. 10.1186/s40168-024-01749-5 38365714 PMC10874076

[B71] Zhang T. Sun P. Geng Q. Fan H. Gong Y. Hu Y. (2022). Disrupted spermatogenesis in a metabolic syndrome model: The role of vitamin A metabolism in the gut-testis axis. *Gut* 71 78–87. 10.1136/gutjnl-2020-323347 33504491 PMC8666830

[B72] Zhang X. Yu D. Wu D. Gao X. Shao F. Zhao M. (2023). Tissue-resident *Lachnospiraceae* family bacteria protect against colorectal carcinogenesis by promoting tumor immune surveillance. *Cell Host Microbe* 31:418–432.e418. 10.1016/j.chom.2023.01.013 36893736

