# Supplementary material for: Metagenomic insights into the effects of Chive seed flavonoid on intestinal fermentation, morphology, and microbiota composition in sheep
Source: Front Microbiol. 2025 Jun 13;16:1590400. doi: 10.3389/fmicb.2025.1590400 (PMC12202629; doi:10.3389/fmicb.2025.1590400)
Supplement: Supplementary file 1 [file Table_1.docx]

**Table S1: All samples sequencing data processing and quality control statistics table (n=60)**

| **Sample ID** | **Sample** | **Input** | **Filtered** | **Denoised** | **Non-chimeric** | **Non-singleton** |
| --- | --- | --- | --- | --- | --- | --- |
| SE_CK_1 | Du_CK_1 | 65399 | 43042 | 42766 | 40675 | 38194 |
| SE_CK_2 | Du_CK_2 | 69938 | 66687 | 66395 | 64698 | 62569 |
| SE_CK_3 | Du_CK_3 | 67581 | 64568 | 64312 | 63057 | 61470 |
| SE_CK_4 | Du_CK_4 | 70556 | 67385 | 67112 | 65842 | 64449 |
| SE_CK_5 | Du_CK_5 | 67521 | 64480 | 64271 | 62605 | 60815 |
| SE_T_1 | Du_T_1 | 70813 | 67643 | 67470 | 67189 | 66342 |
| SE_T_2 | Du_T_2 | 71117 | 68017 | 67803 | 67480 | 66433 |
| SE_T_3 | Du_T_3 | 70361 | 67161 | 66958 | 66633 | 65639 |
| SE_T_4 | Du_T_4 | 66903 | 63834 | 63593 | 63309 | 62310 |
| SE_T_5 | Du_T_5 | 65308 | 43247 | 43064 | 42774 | 41855 |
| KC_CK_1 | Je_CK_1 | 63501 | 42043 | 41851 | 40654 | 38081 |
| KC_CK_2 | Je_CK_2 | 65541 | 43414 | 43193 | 41954 | 39160 |
| KC_CK_3 | Je_CK_3 | 66409 | 43692 | 43409 | 41072 | 38353 |
| KC_CK_4 | Je_CK_4 | 68172 | 45116 | 44904 | 43124 | 40864 |
| KC_CK_5 | Je_CK_5 | 63978 | 42133 | 41912 | 40338 | 38325 |
| KC_T_1 | Je_T_1 | 69932 | 66736 | 66337 | 61812 | 57225 |
| KC_T_2 | Je_T_2 | 69279 | 45633 | 45402 | 43113 | 40103 |
| KC_T_3 | Je_T_3 | 65953 | 63111 | 62804 | 59883 | 56688 |
| KC_T_4 | Je_T_4 | 66242 | 63316 | 63000 | 60268 | 56180 |
| KC_T_5 | Je_T_5 | 66820 | 63886 | 63522 | 60875 | 56741 |
| HC_CK_1 | Il_CK_1 | 66895 | 44155 | 43941 | 42463 | 39625 |
| HC_CK_2 | Il_CK_2 | 65075 | 42917 | 42704 | 40920 | 37798 |
| HC_CK_3 | Il_CK_3 | 69183 | 45526 | 45207 | 43390 | 40181 |
| HC_CK_4 | Il_CK_4 | 66478 | 43706 | 43311 | 40575 | 34788 |
| HC_CK_5 | Il_CK_5 | 66604 | 43745 | 43528 | 41123 | 38262 |
| HC_T_1 | Il_T_1 | 66311 | 63367 | 62832 | 56974 | 49363 |
| HC_T_2 | Il_T_2 | 65969 | 63116 | 62651 | 56746 | 49914 |
| HC_T_3 | Il_T_3 | 68597 | 65643 | 65167 | 58926 | 52371 |
| HC_T_4 | Il_T_4 | 68433 | 65509 | 64972 | 59143 | 52956 |
| HC_T_5 | Il_T_5 | 68588 | 65584 | 65099 | 59431 | 53359 |
| MC_CK_1 | Ce_CK_1 | 63667 | 42949 | 42547 | 40341 | 21819 |
| MC_CK_2 | Ce_CK_2 | 64787 | 43767 | 43379 | 41375 | 22391 |
| MC_CK_3 | Ce_CK_3 | 69088 | 46704 | 46249 | 44022 | 23546 |
| MC_CK_4 | Ce_CK_4 | 66325 | 45008 | 44604 | 42480 | 22859 |
| MC_CK_5 | Ce_CK_5 | 66557 | 44887 | 44421 | 41984 | 22826 |
| MC_T_1 | Ce_T_1 | 67942 | 45840 | 45400 | 43526 | 19465 |
| MC_T_2 | Ce_T_2 | 65356 | 44024 | 43611 | 41607 | 18756 |
| MC_T_3 | Ce_T_3 | 65749 | 44366 | 43914 | 42034 | 19800 |
| MC_T_4 | Ce_T_4 | 63109 | 42569 | 42080 | 40297 | 19909 |
| MC_T_5 | Ce_T_5 | 62670 | 42377 | 42029 | 40225 | 22223 |
| JC_CK_1 | Co_CK_1 | 65130 | 44043 | 43618 | 41898 | 22337 |
| JC_CK_2 | Co_CK_2 | 69426 | 66466 | 65734 | 62657 | 33919 |
| JC_CK_3 | Co_CK_3 | 62684 | 42368 | 41922 | 40416 | 19788 |
| JC_CK_4 | Co_CK_4 | 63798 | 43331 | 42876 | 41235 | 19635 |
| JC_CK_5 | Co_CK_5 | 66808 | 45256 | 44807 | 42928 | 21564 |
| JC_T_1 | Co_T_1 | 67613 | 45779 | 45393 | 42897 | 23236 |
| JC_T_2 | Co_T_2 | 67519 | 45675 | 45230 | 42763 | 23223 |
| JC_T_3 | Co_T_3 | 65644 | 44311 | 43925 | 41809 | 20398 |
| JC_T_4 | Co_T_4 | 64000 | 43342 | 42896 | 40656 | 20715 |
| JC_T_5 | Co_T_5 | 63764 | 43115 | 42729 | 40730 | 21583 |
| ZC_CK_1 | Re_CK_1 | 68112 | 65168 | 64677 | 60275 | 49456 |
| ZC_CK_2 | Re_CK_2 | 64916 | 62234 | 61790 | 57766 | 47848 |
| ZC_CK_3 | Re_CK_3 | 64309 | 43756 | 43342 | 41220 | 34863 |
| ZC_CK_4 | Re_CK_4 | 65250 | 62526 | 62015 | 59399 | 53445 |
| ZC_CK_5 | Re_CK_5 | 64952 | 62251 | 61751 | 58871 | 50956 |
| ZC_T_1 | Re_T_1 | 71084 | 67984 | 67385 | 62215 | 42996 |
| ZC_T_2 | Re_T_2 | 65252 | 43954 | 43569 | 40766 | 27648 |
| ZC_T_3 | Re_T_3 | 67703 | 45634 | 45214 | 42066 | 28339 |
| ZC_T_4 | Re_T_4 | 66255 | 44831 | 44455 | 41668 | 29471 |
| ZC_T_5 | Re_T_5 | 63806 | 42994 | 42616 | 39798 | 28355 |
